# 1268. *In Vitro* Activity of Ceftazidime-Avibactam and Comparators against KPC-Producing Enterobacterales and *Pseudomonas aeruginosa* Collected in China as Part of the ATLAS Global Surveillance Program in 2019

**DOI:** 10.1093/ofid/ofab466.1460

**Published:** 2021-12-04

**Authors:** Mark G G Wise, Krystyna Kazmierczak, Gregory Stone, Daniel F Sahm

**Affiliations:** 1 IHMA, Schaumburg, Illinois; 2 IHMA, Inc., Schaumburg, Illinois; 3 Pfizer, Inc., Groton, CT

## Abstract

**Background:**

Among Gram-negative bacteria, the rapid spread of carbapenemases has limited therapeutic options. *Klebsiella pneumoniae* carbapenemase (KPC), an Ambler class A serine β-lactamase, presents a particular challenge as it has become widespread, first identified in an isolate collected in the United States and thereafter moving throughout the world, including China. Fortunately, the β-lactamase inhibitor avibactam is a potent inhibitor of KPC, rendering many Enterobacterales and some *P. aeruginosa* isolates that carry KPC susceptible to ceftazidime-avibactam (CAZ-AVI) *in vitro*. This study reports on the *in vitro* activity of CAZ-AVI and comparators against Enterobacterales and *P. aeruginosa* isolates collected in China as part of the Antimicrobial Testing Leadership and Surveillance (ATLAS) program in 2019.

**Methods:**

1,443 non-duplicate Enterobacterales and 522 *P. aeruginosa* isolates were collected from 17 clinical sites in China in 2019. Susceptibility testing was done using broth microdilution according to CLSI guidelines and interpreted using CLSI 2021 breakpoints. 143/177 meropenem non-susceptible Enterobacterales isolates and 150/187 meropenem non-susceptible *P. aeruginosa* isolates were interrogated by whole genome sequencing (WGS; Illumina 2x150 bp reads).

**Results:**

Enterobacterales isolates exhibited higher % susceptibility (% S) to CAZ-AVI than all comparators tested (96.0% S; Table). The addition of AVI to CAZ resulted in an increase in susceptibility from 61.3% to 96.0% in the overall collection of Enterobacterales isolates. 96.0% of KPC-positive Enterobacterales, and 67.8% of the meropenem non-susceptible sub-population were susceptible to CAZ-AVI, against which comparators were less active (≤42.9 % S). Among *P. aeruginosa* isolates, 89.8% were susceptible to CAZ-AVI, more than for any comparator except amikacin (AMK; 94.4% S). Against meropenem non-susceptible and KPC-carrying *P. aeruginosa* sub-populations more were susceptible to CAZ-AVI (75.9% and 83.3% S, respectively) and AMK (87.2% and 100% S, respectively) than to other comparators (≤40.6% and ≤8.3% S, respectively).

Results Table

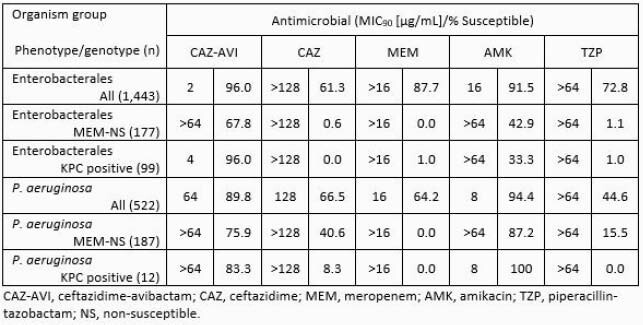

**Conclusion:**

CAZ-AVI demonstrated very good *in vitro* activity against Enterobacterales and *P. aeruginosa* isolates from China, including those that harbor KPC.

**Disclosures:**

**Mark G G. Wise, PhD**, **IHMA** (Employee)**Pfizer, Inc.** (Independent Contractor) **Krystyna Kazmierczak, PhD**, **IHMA** (Employee)**Pfizer, Inc.** (Independent Contractor) **Gregory Stone, PhD**, **AztraZeneca** (Shareholder, Former Employee)**Pfizer, Inc.** (Employee) **Daniel F. Sahm, PhD**, **IHMA** (Employee)**Pfizer, Inc.** (Independent Contractor)

